# Predictors of esophageal varices in patients with HBV-related cirrhosis: a retrospective study

**DOI:** 10.1186/1471-230X-9-11

**Published:** 2009-02-05

**Authors:** Wan-dong Hong, Qi-huai Zhu, Zhi-ming Huang, Xiang-rong Chen, Zen-cai Jiang, Si-hao Xu, Kunlin Jin

**Affiliations:** 1 Department of Gastroenterology and Hepatology, the First Affiliated Hospital of Wenzhou Medical College, Wenzhou, PR China; 2 Department of General Surgery, the Second Affiliated Hospital of Wenzhou Medical College, Wenzhou, PR China; 3 Department of Ultrasound, the First Affiliated Hospital of Wenzhou Medical College, Wenzhou, PR China; 4 Buck Institute for Age Research, Novato, California, USA

## Abstract

**Background:**

All patients with liver cirrhosis are recommended to undergo an evaluation of esophageal varices (EV) to assess their risk of bleeding. Predicting the presence of EV through non-invasive means may reduce a large number of unnecessary endoscopies. This study was designed to develop a predictive model for varices in patients with Hepatitis B virus-related cirrhosis.

**Methods:**

The retrospective analysis was performed in 146 patients with Hepatitis B virus-related cirrhosis. The data were assessed by univariate analysis and a multivariate logistic regression analysis. In addition, the receiver operating characteristic curves were also applied to calculate and compare the accuracy of the model and other single parameters for the diagnosis of esophageal varices.

**Results:**

We found the prevalence of EV in patients with Hepatitis B virus-related cirrhosis to be 74.7%. In addition, platelet count, spleen width, portal vein diameter and platelet count/spleen width ratio were significantly associated with the presence of esophageal varices on univariate analysis. A multivariate analysis revealed that only the spleen width and portal vein diameter were independent risk factors. The area under the receiver operating characteristic curve of regression function (RF) model, which was composed of the spleen width and portal vein diameter, was higher than that of the platelet count. With a cut-off value of 0.3631, the RF model had an excellent sensitivity of 87.2% and an acceptable specificity of 59.5% with an overall accuracy of 80.1%.

**Conclusion:**

Our data suggest that portal vein diameter and spleen width rather than platelet count may predict the presence of varices in patients with Hepatitis B virus-related cirrhosis, and that the RF model may help physicians to identify patients who would most likely benefit from screenings for EV.

## Background

Most cirrhotic patients develop esophageal varices, with a lifetime incidence as high as 90% [1]. Approximately one third of cirrhotic patients with esophageal varices (EV) develop an episode of esophageal hemorrhage, and subsequently have high morbidity and mortality [1]. Therefore, early detection and prevention of EV in cirrhotic patients are crucial to minimizing complications. An endoscopy examination is currently considered to be the gold standard. However, screening all patients with endoscopy to guide therapy may significantly increase the cost.

Non-invasive indicators of varices are desired to reduce the need for screening endoscopy in all patients with cirrhosis. Studies shows that platelet count, splenomegaly, platelet count/spleen diameter ratio, advanced Child-Pugh class, serum albumin, and high portal vein diameter measured by ultrasonography may be useful non-invasive predictors of EV for patients with cirrhosis [2-4]. Such predictive factors may be expected to vary in different populations because of differences in the etiologies of liver cirrhosis and severity of liver disease. Data on this aspect in Chinese patients with liver cirrhosis, who usually have a higher proportion with hepatitis B viral etiology, remain largely unexplored. Here in this retrospective study, we evaluated the utility of various clinical, biochemical and ultrasonographic parameters in developing a model for predicting the presence of EV in Chinese patients with HBV-related cirrhosis.

## Methods

This study included a total of 146 patients with HBV (Hepatitis B virus)-related cirrhosis that attended at our hospital between July 2005 and July 2007. Exclusion criteria included: active bleeding; current alcohol intake (patients with alcohol-related liver cirrhosis were excluded even if abstinent for at least 6 months prior to endoscopy); previous endoscopic sclerosis or band ligation of EV; previous surgery for portal hypertension or transjugular intrahepatic porto-systemic stent shunt placement. None of the patients were treated with β-blocker or diuretics. Hepatocellular carcinoma, spontaneous bacterial peritonitis, or portal vein thrombosis were also excluded for the study.

The following information was collected for each patient: age, gender, biochemical parameters including aspartate aminotransferase (AST), alanine aminotransferase (ALT), ALT/AST ratio, total bilirubin, conjugated bilirubin, total protein, serum albumin, prothrombin time, prothrombin activity (%), alkaline phosphatase, γ-glutamyl transpeptidase (GT), platelet count, presence and degree of ascites and encephalopathy assessed according to Child-Pugh criteria [5]. Diuretics therapy was not commenced before endoscopy and ultrasonography was performed. The presence and size of EV were determined and recorded for each patient. The size of varices was subdivided into two classes–small and large according to the criteria proposed at the Baveno I Consensus Conference [6]. Small EV were defined as varices that flatten with insufflation or minimally protrude into the esophageal lumen, while large EV were defined as varices that protrude into the esophageal lumen and touch each other (presence of confluence), or that fill at least 50% of the esophageal lumen. This study protocol was approved by the Ethic Committee of the First Affiliated Hospital of Wenzhou Medical College.

All patients underwent ultrasonographic examination of the upper abdomen including measurement of spleen width and portal vein diameter. The platelet count/spleen width ratio in all patients was then calculated. Ultrasonographic measurement of spleen width on a longitudinal section with the patient in the right lateral decubitus position was technically feasible in all patients as described by Lamb et al [7]. The intra- and inter-observer coefficients of variation for measuring the spleen width were evaluated in 50 patients and were 1.2% and 2.1%, respectively.

All data were analyzed by STATA 10.0 software. Continuous values were expressed by mean ± SD or median and interquartile range and compared using the Student's t-test or the Mann-Whitney non-parametric test. Categorical values were described by count and proportions and compared by the χ^2 ^test. Univariate analyses for determining the association of various clinical, laboratories and ultrasonographic variables with the presence or absence of EV were performed, and P-values below 0.05 were considered significant. All variables that were found to be different between patients with and without EV on univariate analysis were included as candidate variables in a forward-conditional step-wise logistic regression analysis to identify independent predictors for the presence of such varices. For this analysis, the conditional probabilities for stepwise entry and removal of a factor were 0.05 and 0.10, respectively. Based on the results of multiple logistic regression analysis, a logistic regression equation was developed to predict EV. The receiver operating characteristic curves (ROC curves) were applied to calculate and compare various predictors for the diagnosis of EV. The validity of the model was measured by means of the area under receiver operating characteristic curve (AUROC). A model with an AUROC above 0.7 was considered useful, while an AUROC between 0.8 and 0.9 indicated excellent diagnostic accuracy [4]. The optimum cut-off value was chosen as the value corresponding with the highest accuracy (minimal false sensitivity and false positive results) for single variables, and various cut-off values were investigated for the model to determine the optimal cut-off values for predicting or excluding EV. The sensitivity (Se), specificity (Sp), negative predictive value (NPV), positive predictive value (PPV), and positive likelihood ratio (+LR), negative likelihood ratio (-LR) and diagnostic accuracy (DA) were calculated for various corresponding cut-off values.

## Results

### Patient characteristics

Of the 146 patients included in the study, 99 (67.8%) were male, with a mean age of 53.1 ± 12.2 years. Only seven patients received antiviral treatment. The demographic and clinical characteristics of the patients are shown in Table 1. EV were found in 109 patients (74.7%) and large varices in 41 patients (28.1%). Prevalence of all varices was 67%, 78%, and 75% in patients with Child-Pugh class A, B and C cirrhosis respectively. As Child-Pugh class was categorized into binary variable: Child-Pugh class A versus advanced Child-Pugh class (Child-Pugh class B or C), the prevalence of all varices in patients with Child-Pugh class A was lower than that in patients with advanced Child-Pugh class (67% versus 77%), which, however, did not reach statistical significance (*P *= 0.25). Ascites was found in 60.3% of patients by ultrasonography and clinical examination. The mean portal vein diameter of all patients was 12.6 ± 1.9 mm (range from 9 to 26 mm).

**Table 1:** Demographic and Clinical Characteristics of 146 patients

Characteristic	Data
Age (yr)	53.1 ± 12.2
	
Male (%)	67.8
Child-Pugh score	8(6–9)
Child-Pugh A/B/C (%)	25.4/50.0/24.6
	
Total Bilirubin (μmol/L)	45.5 ± 55.7
	
AST (IU/L)	80.5 ± 70.8
ALT (IU/L)	61.8 ± 66.9
Albumin (g/L)	31.8 ± 6.3
	
Prothrombin time (s)	18.4 ± 4.5
Platelets (10^9^/L)	61.5 ± 37.9
Portal vein diameter (mm)	12.6 ± 1.9
	
Spleen width (mm)	50.6 ± 10.1

Data are shown either as number of observations, percentage or median ± SD except for Child-Pugh score (expressed as median and interquartile range)

### Variables associated with the presence of EV by univariate analysis

Twenty variables considered relevant to the presence of EV were tested using univariate analysis. As shown in Table 2, platelet count, spleen width, platelet count/spleen width ratio, and portal vein diameter were significantly associated with the presence of EV.

**Table 2:** Univariate analysis of predictive factors of EV in 146 patients

Variable	Absence of varices N = 37	Presence of varices N = 109	P
Age (yr)	54.8 ± 13.6	52.5 ± 11.7	0.327
Male (%)	75.7	65.1	0.236
Child-Pugh score	8(6–9)	8(7–9)	0.789
Child-Pugh class			0.487
A	12(33%)	25(23%)	
B	16(43%)	57(52%)	
C	9(24%)	27(25%)	
Total Bilirubin (μmol/L)	41.4 ± 40.7	46.9 ± 60.0	0.607
			
Conjugated bilirubin(μmol/L)	18.2 ± 21.0	20.6 ± 34.9	0.988
			
Total protein (g/L)	64.1 ± 5.3	63.2 ± 7.2	0.467
Albumin (g/L)	31.3 ± 6.2	31.9 ± 6.3	0.596
ALT (U/L)	75.6 ± 95.8	57.0 ± 51.8	0.139
AST (U/L)	91.5 ± 99.1	76.7 ± 58.3	0.273
ALT/AST ratio	0.79 ± 0. 33	0. 77 ± 0. 27	0.719
			
Alkaline phosphatase (U/L)	109.4 ± 41.0	108.9 ± 70.9	0.969
			
γ- GT (U/L)	115.6 ± 139.1	82.3 ± 94.6	0.106
Prothrombin time(s)	18.7 ± 5.5	18.3 ± 4.1	0.691
			
Prothrombin activity (%)	60.4 ± 17.9	60.7 ± 15.2	0.925
Platelets (10^9^/L)	79.6 ± 51.1	55.3 ± 30.1	< 0.001
			
Ascites (%)	48.6	64.2	0.094
Platelets/Spleen width	1.89 ± 1.39	1.15 ± 0.82	< 0.001
			
Spleen width (mm)	44.8 ± 7.6	52.5 ± 10.1	< 0.001
			
Portal vein diameter (mm)	11.6 ± 1.3	12.9 ± 1.9	< 0.001

Data are shown either as percentage or median ± SD except for Child-Pugh score (expressed as median and interquartile range).

### Factors independently associated with EV

A multivariate analysis was performed by a logistic regression for all variables. As shown in Table 3, significant independent association with the presence of EV was found for the spleen width (p = 0.022) and portal vein diameter (p = 0.009). In addition, a regression equation was derived for predicting the presence of esophageal varices. Regression function (RF) = -8.107529 +0.4758 (portal vein diameter) + 0.0708 (spleen width). The *P*-value of this regression function is < 0.0001. We denominated it as the RF model. The Hosmer-Lomeshow goodness of fit test was significant (*p *= 0.4282), suggesting that our prediction model fit the actual data well.

**Table 3:** Logistic regression model to predict the presence of EV in 146 patients

Variable	Coefficient	Odds Ratio (95% Confidence interval)	P
Constant	-8.107529		
Spleen width (mm)	0.0708089	1.07 (1.01–1.14)	0.022
Portal vein diameter (mm)	0.4757532	1.61 (1.13–2.29)	0.009

### Diagnostic values of RF model, portal vein diameter, spleen width, platelet count and platelet count/spleen width ratio

**Figure 1:**
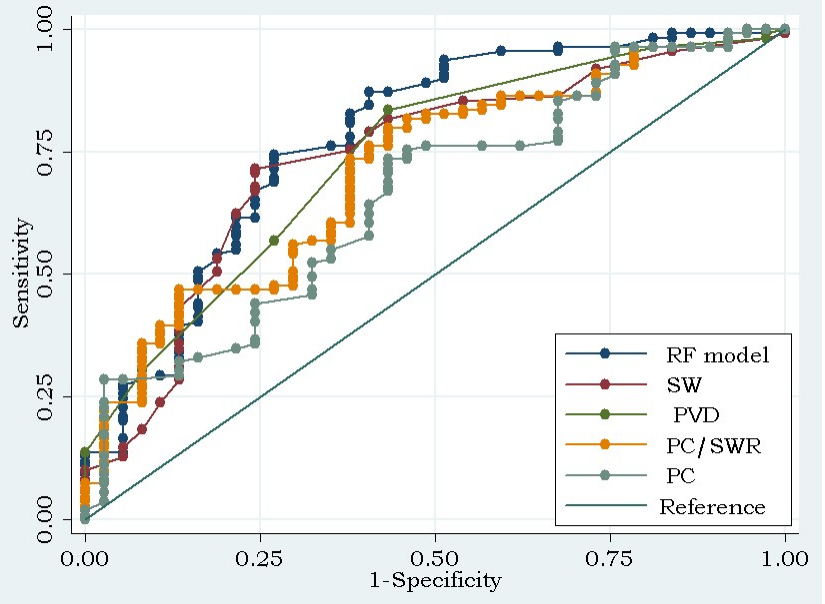
**Receiver operating characteristic curves for various predictors of EV**. The areas under receiver operating characteristic curves were 0.777 ± 0.048, 0.739 ± 0.047, 0.736 ± 0.049, 0.7095 ± 0.0488, 0.660 ± 0.052 for the RF model, portal vein diameter (PVD), spleen width (SW), platelet count/spleen width ratio (PC/SWR) and platelet count (PC) respectively. The ideal area under the curve was 1.00. The reference line represents that based on chance alone (area under the curve 0.50).

The receiver operating characteristic (ROC) curve for RF model, portal vein diameter, spleen width, platelet count and platelet count/spleen width ratio for the prediction of EV are shown in Figure 1. The RF model yielded the highest AUROC (0.777 ± 0.048) and the AUROC of it was statistically higher than that of the platelet count (AUROC = 0.660 ± 0.052, P = 0.0252) by a significant amount. However, the AUROC of the RF model was not substantially different from the AUROC of portal vein diameter (AUROC = 0.739 ± 0.047, P = 0.08), spleen width (AUROC = 0.736 ± 0.049, *p *= 0.12), or platelet count/spleen width ratio (AUROC = 0.7095 ± 0.0488, p = 0.10). The AUROCs of the RF model, portal vein diameter, spleen width, platelet count/spleen width ratio and platelet count were all > 0.5 statistically.

Based on the ROC curve analysis, the optimum cut-off values of portal vein diameter, spleen width, and platelet count/spleen width ratio were 12 mm, 46 mm, 1.0153 (10^9^/L)/mm, respectively (Table 4). The diagnostic performances for different cut-off values of the RF model are shown in Table 5.

**Table 4:** Performance of the portal vein diameter, spleen width and the platelet count/spleen width ratio for predicting the presence of EV.

Variable	Best Cut-off	Se (%)	Sp (%)	+LR	-LR	PPV (%)	NPV (%)	DA (%)
Portal vein diameter (mm)	12	83.5	56.8	1.93	0.29	85.1	53.8	76.7
Spleen width (mm)	46	71.6	75.7	2.94	0.38	89.7	47.5	72.6
Platelet count/Spleen width	1.0513	57.8	67.6	1.78	0.62	84.0	35.2	60.3

Se, sensitivity; Sp, specificity; +LR, positive likelihood ratio; -LR, negative likelihood ratio; PPV, positive predictive value; NPV, negative predictive value; DA, diagnostic accuracy; Prevalence of esophageal varices, 74.7% (109/146)

**Table 5:** Diagnostic values of RF model for predicting EV at various cut-off points.

Cut-off	Se (%)	Sp (%)	+LR	-LR	PPV (%)	NPV (%)	DA (%)
-03052	99.1	16.2	1.18	0.06	77.1	85.7	77.4
0.0997	95.4	40.5	1.60	0.11	82.6	74.8	81.5
0.1705	93.6	48.7	1.82	0.13	84.3	72.0	82.2
0.2214	90.8	48.6	1.79	0.17	83.9	64.1	80.8
0.3631	87.2	59.5	2.15	0.22	86.4	61.2	80.1
0.5445	82.6	62.2	2.18	0.28	86.6	54.8	77.4
0.7879	74.3	73.0	2.75	0.35	89.0	49.0	73.3
1.6177	50.0	83.8	3.11	0.59	90.2	36.5	60.3
3.014	13.8	100	0	0.86	100	38.2	35.6

Se, sensitivity; Sp, specificity; +LR, positive likelihood ratio; -LR, negative likelihood ratio; PPV, positive predictive value; NPV, negative predictive value; DA, diagnostic accuracy; Prevalence of esophageal varices, 74.7% (109/146);

## Discussion

Thrombocytopenia in patients with cirrhosis has historically been attributed to hypersplenism due to portal hypertension. Several studies suggest that platelet count may predict the presence of EV in patients with cirrhosis [2,8,9]. However, the discriminating threshold for the presence of varices varies widely, ranging between 68,000 and 160,000/mm^3 ^[10]. The sensitivities for thrombocytopenia fluctuate from 62% to 100%, and the specificities range from 18% to 77% [11]. Our data suggest that the multivariate analysis failed to show any significant difference between thrombocytopenia and the risk of EV. In addition, platelet count might not be an ideal predictor of the presence of EV in HBV-related cirrhosis. A possible explanation is that other factors, such as suppressive effects of viruses on bone marrow and antibody-mediated destruction of platelets, may play a more important role in HBV-related cirrhosis than that in alcohol cirrhosis, in addition to decreased thrombopoeitin and interleukin-11 [12].

The prevalence of EV in this study is similar to previous findings [13]. In addition, the prevalence of all varices in patients with advanced Child-Pugh class was higher than that in patients with Child-Pugh class A, which is also consistent with previous observations that patients with advanced liver disease are more likely to have varices [8]. However, this difference did not reach statistical significance, which could be due to the small sample size of our study. Data on the relationship between Child-Pugh score and the risk of EV is somewhat conflicting. One study showed that Child-Pugh score was an acceptable predictor of the presence of EV [14], whereas a recent well-designed study suggested that it was not a useful predictor [15]. Our data do not support any correlation between Child-Pugh score with varices, which may partially be due to a rather homogeneous population in the present study, in which 50% of patients were Child-Pugh class B. As suggested by Rajvanshi et al, the prevalence of EV increased with a higher Child-Pugh class, but Child-Pugh score was not a predictor of EV [16].

EV is the direct consequence of spontaneous formation of collateral vessels between portal vein and esophageal veins via left gastric or short gastric veins. Therefore, the presence or absence of EV may reflect the severity of portal hypertension. In a logistic regression study of 143 patients [17], ultrasonographic portal vein diameter greater than 13 mm was one of the independent risk factors for the presence of EV. However, another study [3] failed to confirm the predicting role of portal vein diameter when using a cut-off value of 13 mm in prevalently HCV-related cirrhosis. The results of the present study indicate that the portal vein diameter with an AUROC of 0.739 could be a valuable predictor of EV in patients with HBV-related cirrhosis. The fact that different studies conclude different best cut-off values of the portal vein diameter may be explained at least in part by that the physique of Asian populations is smaller than that of European populations. It has been reported that the normal mean portal vein diameter in Chinese populations is 9.5 ± 1.3 mm [18], while it is 11.0 ± 0.3 mm in French populations [19].

Splenomegaly is recognized as one of the diagnostic signs of cirrhosis and portal hypertension. Several studies show that splenomegaly may be a good predictor of EV [2,9,20,21]. Lamb et al found that there was a good correlation between in vivo ultrasound assessment of splenic width and true splenic volume [7]. Our data showed that spleen width measured by ultrasonography was an independent predictor for the presence of varices with an AUROC of 0.736. Contrary to what was suggested in previous reports, no correlation between splenomegaly and EV was found in other studies [17,22,23]. These differences may be due to the variations among studies regarding the etiology and the stage of liver cirrhosis studied. Moreover, splenomegaly is found more frequently in posthepatitic cirrhosis than in alcoholic cirrhosis [24]. With the best cut-off value of 1.0513 (10^9^/L)/mm, the platelet count/spleen width ratio yielded a low diagnostic accuracy of 60.3%, which suggests that it is not an ideal predictor for EV.

With a cut-off value of 0.3631, the RF model had an excellent sensitivity of 87.2% and an acceptable specificity of 59.5% with an overall accuracy of 80.1%, which was higher than that of spleen width and portal vein diameter. And with a cut-off value of 0.1705, 82.2% of the patients were correctly classified. However, our data showed that the RF model had a moderate predictive power with an AUROC of 0.777. Moreover, the other parameters listed in Table 5 are also modest: the positive likelihood ratios of the different cut-off values are rather low, and the negative likelihood ratios are too high except for the cut-off -0.3052. However, the specificity for the cut-off -0.3052 is fairly low, which means that some of these patients with splenomegaly and dilated portal vein may not have EV. One of the possible explanations for this result could be the development of spontaneous intra-abdominal shunts that decrease the blood flow of varices while maintaining congestive splenomegaly and dilated portal vein.

Our study has several limitations. First, the data were collected retrospectively, so patients at a relatively more advanced stage of the disease were more prone to be enrolled in our study, while outpatients were more likely to be excluded for insufficient data, which may produce a population bias. Second, prediction models may vary with the nature of the patient population from which these are derived. The population of our study is largely composed of patients with decompensated cirrhosis, and only partially reflects the population in which an index to predict the presence of varices would be mostly applied in clinical practice, i.e. patients enrolled at the time of the diagnosis of cirrhosis. This might have artificially increased the performance of the prognostic index. The predictability of variables in the RF model might not apply to a patient or cohort with less advanced cirrhosis. Prospective validation in an independent patient population is mandatory. Whereas the authors have to state clearly that until this is done, the model should not be used as a substitute for endoscopy because the parameters of the model are all modest. Third, the performance characteristics of the index is globally modest – the AUROC is inferior to that of other indexes such as the platelet count/spleen diameter ratio [4,25,26], CT esophagography [27], all of which are recently considered as excellent predictors for EV. It will be interesting and necessary to analyze these predictors in comparison in future studies.

Although the present study has the above-mentioned limitations, it is the first study, to the best of our knowledge, to investigate predictors of EV in HBV-related cirrhosis in homogeneous population. In addition, the platelet count/spleen width ratio and the spleen width on ultrasonography were first introduced as potential predictors of EV. The intra- and inter-observed variability for the spleen width measurement is good, so it is reproducible. The RF model with a certain cut-off value has a much higher sensitivity and NPV for ruling out the presence of EV and higher diagnostic accuracy than that of single predictors such as the platelet count/spleen width ratio, the spleen width, the portal vein diameter.

## Conclusion

Portal vein diameter and spleen width but not platelet count may predict the presence of varices in hepatitis B virus-related cirrhosis. The RF model may also help clinicians to identify patients who would most likely benefit from referrals for screening for esophageal varices. However, these findings, including the validity of the model, need to be verified with prospectively collected data. Endoscopic screening for EV may be recommended in HBV-related cirrhotic patients with splenomegaly and dilated portal vein.

## Competing interests

The authors declare that they have no competing interests.

## Authors' contributions

QHZ and ZMH joined in the design of the study. WDH, SHX and ZCJ carried out the studies, participated in the sequence alignment and data collection. WDH conducted data analysis and drafted the manuscript. XRC and KJ supervised to complete the data analysis and helped to finalize the manuscript. All of the authors read and approve the manuscript.

## Pre-publication history

The pre-publication history for this paper can be accessed here:

http://www.biomedcentral.com/1471-230X/9/11/prepub
